# GANAB and PKD1 Variations in a 12 Years Old Female Patient With Early Onset of Autosomal Dominant Polycystic Kidney Disease

**DOI:** 10.3389/fgene.2019.00044

**Published:** 2019-02-07

**Authors:** Elizabeth Waldrop, Mohammed A. I. Al-Obaide, Tetyana L. Vasylyeva

**Affiliations:** Department of Pediatrics, Texas Tech University Health Sciences Center, Amarillo, TX, United States

**Keywords:** autosomal dominant polycystic kidney disease, glucosidase II alpha subunit, polycystin-1, nonsense mutation, missense mutation, transcription factors binding sites

## Abstract

Autosomal Dominant Polycystic Kidney Disease (ADPKD) typically results from a mutation in the PKD1 and PKD2 genes, which code for polycystin-1 (PC1) and polycystin-2 (PC2), respectively. Mutations in these genes promote renal cystic dysplasia and are a significant cause of End-Stage Kidney Disease (ESKD). Polycystic kidney disease-3 (PKD3), another form of ADPKD, is caused by mutations in glucosidase II alpha subunit (GANAB) gene and present in mid- and late adulthood. We report a description of an ADPKD case in a 12-year-old female presented bilateral renal cysts in adolescence. Two mutations in two genes PKD1 and GANAB were identified by targeted capture and next-generation sequencing (NGS) on an Illumina sequencing system. The identified PKD1 mutation p.Pro61Leu: c.182C > T (CCC > CTC) a missense type of uncertain clinical significance. However, the identified PKD1 mutation can alter transcription factors motifs and consequently disturb the transcription process. The second mutation identified in GANAB locus, p.Arg61Ter: c.181C > T, a nonsense type, CGA > TGA. The mutation is unreported pathogenic variant can cause loss of the glucosidase II alpha subunit normal protein function. Both the patient father and paternal grandmother had a history of ADPKD but never were tested. This case is the first case of combine presentation on PKD1 and PKD3 in a pediatric patient with nephrolithiasis.

## Introduction

Polycystic kidney disease (PKD) is the cause of chronic renal failure in children and adults. The disease is inherited by an autosomal dominant trait (ADPKD) or an autosomal recessive trait (ARPKD) (Igarashi and Somlo, 2002). ADPKD is genetically inherited and caused by mutations in two main genes, PKD1 (80–85%) and PKD2 (15–20%) coding for PC1 and PC2, respectively (Cornec-Le Gall et al., 2013; Cornec-Le Gall et al., 2018). It was shown the age at onset of ESKD was 58 years for PKD1 carriers and 79 years for PKD2 carriers (Cornec-Le Gall et al., 2013). The ADPKD Mutation Database presented 2323 and 278 germline pathogenic and non-pathogenic mutations in PKD1 and PKD2, respectively as of April 23, 2018. Whereas, the reported number of somatic mutations in the ADPKD Mutation Database were 9 and 27 for PKD1 and PKD2, respectively. In about 50% of cases, ADPKD progresses to end-stage renal disease (Braun, 2009). Until recently, mutations in the genes coding for polycystin-1 and polycystin-2 were believed to be the only source of genetic renal cyst formation. In 2016, several mutations in GANAB, encoding the Glucosidase IIα subunit were reported to cause ADPKD (Porath et al., 2016); it was called PKD3 and the only one boy became symptomatic at the age 9 years. The authors showed 20 affected individuals, majority mid-late age adults, from nine families with GANAB mutations. Here, we describe a case of a dual mutation in PKD1 and GANAB genes with an early clinical presentation in a 12-year-old adolescent girl with renal cyst formation and nephrolithiasis; and discuss impacts of dual mutation on the severity of the disease.

## Case Presentation

**FIGURE 1:**
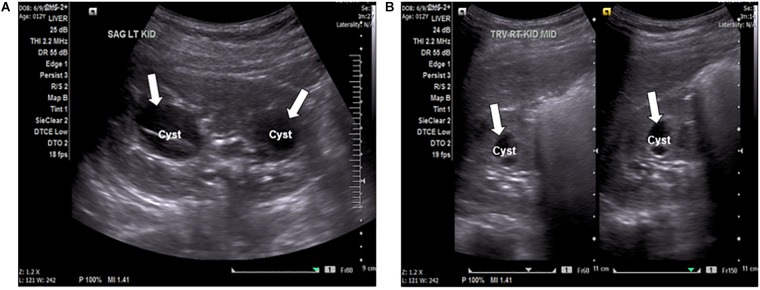
**(A)** Ultrasound of the left kidney, sagittal view, showing anechoic fluid-filled septated cystic spaces (arrows) insinuating the renal sinuses. The cyst on the left is an image of the largest cyst present in the kidneys, measuring 3.3 mm. **(B)** Ultrasound of the right kidney, transverse view, showing anechoic fluid-filled multi-septated spaces (arrows).

A 12-year-old female presented to the local emergency room with persistent intense left flank pain. Dipstick showed large blood and abdominal CT showed 4 mm obstructing calculus in the proximal left ureter, nephrolithiasis with minimal scarring in the upper pole of left kidney, multiple bilateral renal cysts with the dominant on the left kidney at 2.8 mm. Non-calcified 2 mm right lower lobe pulmonary nodules was also identified. Renal function was preserved with the BUN of 11 mg/dl and creatinine of 0.6 mg/dl, electrolytes were within normal range. The patient was treated with pain control medications and hydration with improvement and was referred to a nephrologist. At the nephrology clinic, urine was collected over 24 h for a “stone risk study” and renal ultrasound (RUS) was performed. RUS showed multiple bilateral cysts and renal calculi in the kidneys (Figure 1A,B). The right kidney measured 10.5 cm × 4.9 cm × 4.8 cm and the left kidney measured 9.8 cm × 4.7 cm × 5.0 cm. Renal cysts were present bilaterally with some displaying thick internal septation (Bosniak type II renal cyst). The largest cyst was present in the left kidney, measuring 3.3 mm. There were no solid masses present. An extrarenal pelvis was present on the left. There was no caliectasis present.

Twenty-four hours urine “stone study” showed elevated levels of calcium oxalate, brushite, and monosodium urate. Because of strong family history, both the father and paternal grandmother had a history of never genetically tested ADPKD, and radiological and clinical finding the patient underwent genetic testing for PKD1, PKD2, GANAB, and HNF1B. Using genomic DNA from the submitted specimens, the exonic regions and flanking splice junctions of the genome were captured and sequenced by next-generation sequencing (NGS) on an Illumina sequencing system and compared to the human genome build of non-mutated genes of interest. The results returned positive mutations for GANAB and PKD1. The GANAB mutation was classified as the pathogenic variant and the PKD1 mutation was classified as a variant of uncertain significance.

### Genomic Contexts of GANAB and PKD1 Identified Mutations

**FIGURE 2:**
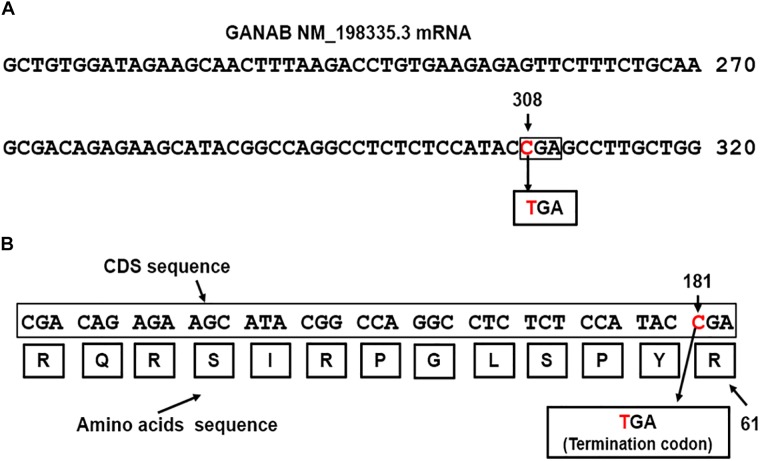
**(A)** The detected nonsense mutation, CGA>TGA, located at nucleotide 308 in the in exon 3 of GANAB NM_198335.3 mRNA (Nucleotide-NCBI/GANAB, 2018). **(B)** Map location of the nonsense mutation in the GANAB-CDS is at position181, p.Arg(R)61Ter: c.181C > T. The mutation causes termination of transcription.

GANAB is vital for Polycystin-1 and Polycystin-2 maturation (Porath et al., 2016). The three genes, GANAB, PKD1, and PKD2 are characterized by a high number of exons, 26, 50 and 16 exons, respectively. We identified dual mutations in GANAB and PKD1genes, which are mapped on the reverse strands on long and short arms of chromosomes 11 and 16, respectively (Gene-NCBI/GANAB, 2018; Gene-NCBI/PKD1, 2018). The identified GANAB mutation resulted in generating nonsense codon that terminates transcription (CGA > TGA) of GANAB mRNA. The mutation located in exon 3 of GANAB mRNA NM_198335.3 (Nucleotide-NCBI/GANAB, 2018). Figure 2A shows the position of the mutated nucleotide C > T in the CGA codon generating nonsense codon TGA at position 308 of GANAB mRNA NM_198335.3. The site of mutation is equivalent to the nucleotide site 181 in the CDS sequence, c.181C > T (Figure 2B). The GANAB-CDS occupies a region of 2900 nucleotides (nts) located at 128–3028 in the GANAB mRNA NM_198335.3. The mutation is predicted to stop transcription of the amino acid arginine (R) coded by CGA at position 61 of the glucosidase II alpha subunit polypeptide, p.Arg(R)61Ter (Figure 2B) and causes loss of normal protein function. It is possible to interpret the GANAB variant as a pathogenic variant, which is not reported in large population cohorts (Lek et al., 2016).

**FIGURE 3:**
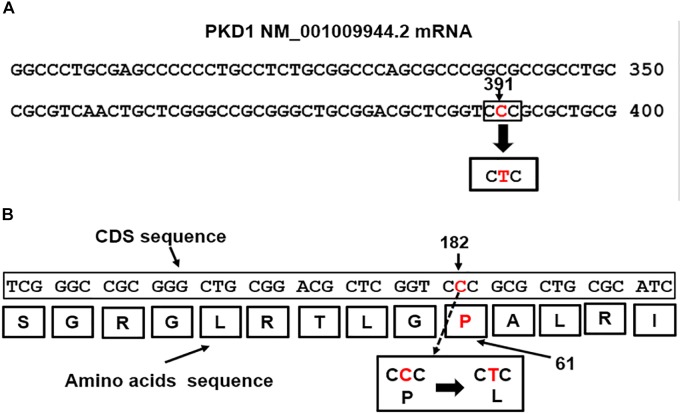
**(A)** The PKD1 missense mutation, CCC>CTC, located at nucleotide 391 in exon1 of the PKD1 NM_001009944.2 mRNA (Nucleotide-NCBI/PKD1, 2018). **(B)** Map location of the missense mutation in the PKD1-CDS is at position182, c.182C > T. The mutation caused replacement of Proline (P) coded by CCC at position 61 in the Polycystin-1 polypeptide by Leucine (L), p.Pro(P)61Leu(L).

Mutations in the PKD1 gene are linked to renal cystic dysplasia and renal tubulogenesis (Qian et al., 2002). The detected PKD1 mutation p.Pro(P)61Leu(L): c.182C > T mapped at nucleotide site 391 in the exon1 of the PKD1-mRNA transcript NM_001009944.2 (Figure 3A) equivalent to position 182 of the CDS sequence (Figure 3B). The mutation is of missense type. The CTC codon is generated from base pair substitution of the middle letter of the CCC codon, CCC>CTC. The triplet CCC codes for proline (P), the mutated codon, CTC, codes for leucine (L) and is predicted to replace proline in the polycystin-1 polypeptide sequence (Figure 3B).

**FIGURE 4:**
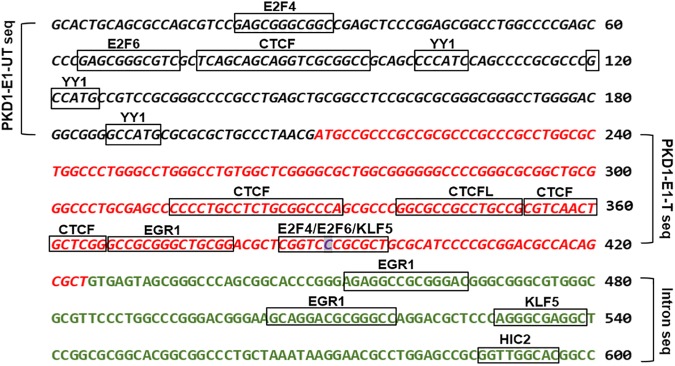
The bioinformatic analysis of PKD1-Exon 1 (E1) untranslated (UT), and translated (T) sequences detected transcription factors binding sites (TFBSs) denoted by boxes. The italic black letters represented PKD1-E1-UT sequence and the red italic letters represented PKD1-E1-T sequence. The green letters refer to the adjacent intron sequence. The highlighted base “C” is the site of mutation. JASPAR 2018 tool was used to detect TFBSs (Khan et al., 2018).

Missense mutations are either silent or mild mutations unless they occur at critical sites along the polypeptide chain. Although, the identified PKD1 missense mutation (p.Pro(P)61Leu(L) is likely of uncertain clinical significance. The finding that showed about 15% of human codons are dual-use codons (duons) specify both amino acids and transcription factor (TF) recognition sites (Stergachis et al., 2013), motivated us to investigate the exon 1 for “duons”. Our analysis showed the PKD1-exon 1 untranslated and translated regions host motifs for eight types of transcription factors (Figure 4 and Supplementary Table S1). Intriguingly, the site of the detected missense mutation showed three overlapping TFs motifs. Also, we identified motifs for two types of genomic structural regulators CTCF and YY1.

## Discussion

Autosomal dominant polycystic kidney disease is the most common hereditary renal disease, occurring in approximately 1 in 400 to 1000 live births (Davies et al., 1991; Torres and Harris, 2009). It is characterized by the focal development of cysts and distortion of kidney architecture, resulting in ESRD in over 50% of patients (Gabow et al., 1992). The clinical severity of cystogenesis and symptomatic disruption of kidney architecture in ADPKD is dependent on the type of genetic mutation, the presence of comorbid conditions, and potentially other factors (Parfrey et al., 1990). Disruption of PKD1 results in ADPKD type 1, characterized by a progressive onset of bilateral renal cysts, cysts present in other organs such as the liver, abdominal wall hernias, and vascular abnormalities (Harris and Torres, 2015). PKD1 variants are often associated with more severe disease and an earlier age of onset with biallelic variations in PKD1 resulting in the even earlier onset of cystogenesis and more severe pathogenic anatomical variations (Harris and Hopp, 2013). Variants in PKD2 are associated with more mild disease progression and later age of onset to ESRD (Fencl et al., 2009). PKD1 and PKD2 are often the only genes tested when evaluating a patient with a positive family history of ADPKD at the patient age of 20–21 years of age or a patient presenting with accidental findings of renal cysts on imaging or symptomatic manifestations of renal cysts, such as nephrolithiasis or hematuria (Cadnapaphornchai, 2015). In 7–10% of patients, the two-gene, PKD1 and PKD2, the analysis does not yield identification of the mutated gene and leaves families with a genetically unresolved cause of their disease (Iliuta et al., 2017). Most of these families do not receive the recommendation to undergo further testing for weak alleles that explain the presence of ADPKD in their lineage, which is supported by the evidence that the weak alleles produce mild to very mild disease and often remains asymptomatic throughout their life (Dicks et al., 2006). However, some non-PKD gene-caused ADPKD results in renal and extra-renal phenotypic manifestations, suggesting that these genes deserve further research to establish their effects in affected families and potential impact on the variations of ADPKD (Besse et al., 2017).

In this study, the identified PKD1 missense mutation caused replacement of proline by leucine. Therefore, it is unlikely the mutation will cause a malfunction in the protein activity. Both the wild and mutant amino acids belong to the hydrophobic group. There are examples to use the 3D structure, physicochemical properties of amino acids, and energy calculations, to envisage the molecular effect due to missense mutation to identify disease-initiating missense mutation (Zhang et al., 2012). Furthermore, the discovery that PKD1-exon1 mutation site within sequence includes transcription factors’ motifs modified by the detected mutation suggested the potential influence on the transcription process. This finding supported by a previous study that showed about 15% of human codons are dual-use codons, which specify both amino acids and TF recognition sites (Stergachis et al., 2013). We speculate, the reported analysis of transcription factor motifs at the sequence of the PKD1 mutation p.Pro(P)61Leu(L): c.182C > T can change transcription process compared to the normal, possibly by interfering with the recruitment of the TFs.

Gene, GANAB, has been recently identified as a cause of ADPKD in 0.3% of patients with the disease (Porath et al., 2016). GANAB encodes glucosidase II subunit a, the catalytic subunit of the protein, and yielded interest due to the proven promotion of cystogenesis in PRKCSH mutations, encoding glucosidase II subunit b, the non-catalytic subunit of the protein (Besse et al., 2017). Mutations in the glucosidase II protein by either mutation resulted in decreased surface and ciliary localization of PC1 and PC2 due to the absolute requirement of the presence of the protein for appropriate localization (Besse et al., 2017). Both mutations in PRKCSH and GANAB were shown to have mild to very mild cystogenic effects in the kidneys and varying effects on cystogenesis in the liver (Porath et al., 2016). In this case, the nonsense GANAB mutation resulted in symptomatic nephrolithiasis resulting in clinical evidence of bilateral renal cysts at the age of 12, contradicting the previous data suggesting the mutation results in subclinical to mild disease.

One of the factors that could affect the clinical progression of the disease as well as prognosis is the presence of additional gene mutations that could promote cystogenesis and augment the advancement toward ESKD (Bergmann et al., 2011). There is evidence that the PKD1 and PKD2 disease loci can have synergistic effects, accelerating the progression to ESRD approximately 20 years earlier than patients with only PKD1 or PKD2 mutation (Bergmann et al., 2011). The clinical progression and disease prognosis in a patient with a mutated PKD1 in combination with a GANAB mutation is unknown but could be potentially synergistic as is the case of dual mutations in PKD1 and PKD2. The results recovered by genetic analysis, in this case, provides novel data suggesting that this mutation in GANAB, outlined in Figure 2, may result in moderate to severe renal cystogenesis. The identification of GANAB mutation provides data for future research in the development of gene therapies targeted for specific mutations. Genetic testing of genes such as GANAB in addition to the genetic testing for PKD1 and PKD2 may yield data encouraging attention toward therapeutic approaches to the management of ADPKD. With the range of clinical manifestations of ADPKD, the testing of patients with rapidly progressive ADPKD may show the presence of additional mutations in weak alleles that may affect the course and severity of their disease (Hopp et al., 2012).

Nephrolithiasis is an important manifestation of ADPKD, which occurs in approximately 20% of patients (Torres et al., 1993) and was one of the clinical manifestations in our patient with the dual mutation. It is not clear whether both mutations play a role in the stone formation or just PKD1 (Bairami et al., 2016). The high incidence of nephrolithiasis is thought to be the consequence of the combination of anatomic abnormality and metabolic risk factors in ADPKD patients, the composition of the stones is most frequently uric acid and/or calcium oxalate (Torres et al., 1993). Metabolic factors are important in their pathogenesis. Distal acidification defects may be important in a few patients, while an abnormal transport of ammonium, low urine pH, and hypocitraturia are the most common abnormalities (Torres et al., 1993).

An intriguing problem reported in ADPKD is an apparent nonexistence of family history suggesting *de novo* mutation in somatic tissues (Reed et al., 2008; Iliuta et al., 2017). PKD Mutation Database^1^ showed nine types of pathogenic somatic variants. Furthermore, mutations of a newly identified gene for, GANAB, and somatic mosaicism need to be considered in the mutation-negative patients with the focal disease. Although there are reports of *de novo* mutations involved in ADPKD (Reed et al., 2008; Iliuta et al., 2017), we assume most of the mutations are not, since the average *de novo* mutation rate is 1.20 × 10^-8^ per nucleotide per generation (Kong et al., 2012). This argument is supported by the prevalence data of ADPKD, which are roughly 1 in 400 to 1 in 1000 live births in all ethnic groups (Torres and Harris, 2009), and a wide range of high phenotypic variability of ADPKD (Cornec-Le Gall et al., 2013).

In summary, the presented case is the first reported pediatric case with dual mutation (PKD1 and GANAB) and nephrolithiasis. The data present unreported novel GANAB mutations to expand the mutation spectrum reported by Porath et al. (2016). Knowledge of spectrum variety in pediatric patients with cystic kidney disease might direct early diagnostic and open prospective for gene therapy.

## Author Contributions

TV, MA-O, and EW conceived the research design of the case study, and wrote, revised, and approved the manuscript. TV and EW examined the patient and recorded the signs and symptoms. MA-O conducted the bioinformatic and genomic analysis.

## Conflict of Interest Statement

The authors declare that the research was conducted in the absence of any commercial or financial relationships that could be construed as a potential conflict of interest.

## Footnotes

1 http://pkdb.mayo.edu/
