# Supplementary material for: GANAB and PKD1 Variations in a 12 Years Old Female Patient With Early Onset of Autosomal Dominant Polycystic Kidney Disease
Source: Front Genet. 2019 Feb 7;10:44. doi: 10.3389/fgene.2019.00044 (PMC6375066; doi:10.3389/fgene.2019.00044)
Supplement: Supplementary file 1 [file Table_1.docx]

Supplementary Material

GANAB and PKD1 Variations in a 12 Years Old Female Patient with Early Onset of Autosomal Dominant Polycystic Kidney Disease

Elizabeth Waldrop^1^, Mohammed A.I. Al-Obaide^1^, Tetyana L. Vasylyeva^1*^

**^1^ Department of Pediatrics, Texas Tech University Health Sciences Center, Amarillo, TX, USA.**

*** Correspondence:** Tetyana L. Vasylyeva: [tetyana.vasylyeva@ttuhsc.edu](mailto:tetyana.vasylyeva@ttuhsc.edu)

**Table S1.** The transcription factors binding sites (TFBSs) for eight types of transcription factors (TF) detected in the PKD1-exon 1 translated and untranslated regions and adjacent intron sequence.

| **TF** | **Start** | **End** | **Strand** | **TFBS Sequence** |
| --- | --- | --- | --- | --- |
| E2F4 | 21 | 31 | + | GAGCGGGCGGC |
| E2F6 | 64 | 74 | + | GAGCGGGCGTC |
| CTCF | 77 | 95 | + | TCAGCAGCAGGTCGCGGCC |
| YY1 | 101 | 106 | + | CCCATC |
| YY1 | 120 | 125 | + | GCCATG |
| YY1 | 187 | 192 | + | GCCATG |
| CTCF | 314 | 332 | -  + | TGGGCCGCAGAGGCAGGGG  RC: CCCCTGCCTCTGCGGCCCA |
| CTCFL | 339 | 352 | -  + | CGGCAGGCGGCGCC  RC: GGCGCCGCCTGCCG |
| CTCF | 353 | 366 | + | CGTCAACTGCTCGG |
| EGR1 | 367 | 380 | -  + | CCGCAGCCCGCGGC  RC: GCCGCGGGCTGCGG |
| E2F4 | 386 | 396 | -  + | GCGCGGGACCG  RC: CGGTCCCGCGC |
| KLF5 | 388 | 397 | + | GTCCCGCGCT |
| E2F6 | 386 | 396 | -  + | GCGCGGGACCG  RC: CGGTCCCGCGC |
| EGR1 | 453 | 466 | -  + | GTCCCGCGGCCTCT  RC: AGAGGCCGCGGGAC |
| EGR1 | 505 | 518 | -  + | GGCCCGCGTCCTGC  RC: GCAGGACGCGGGCC |
| KLF5 | 530 | 539 | -  + | GCCTCGCCCT  RC: AGGGCGAGGC |
| HIC2 | 588 | 596 | -  + | GTGCCAACC  RC: GGTTGGCAC |

**
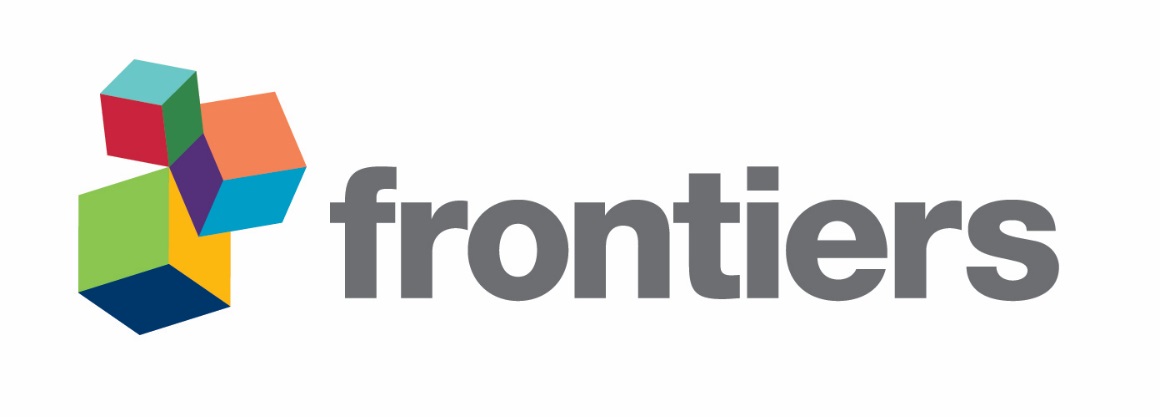
**
